# Topology-Dependent Modeling Fidelity of Lattice-Reinforced UAV Leading-Edge Structures Under Bird Strike Impact

**DOI:** 10.3390/ma19204370

**Published:** 2026-10-17

**Authors:** Muhammad Arslan Muneer, Antonio Garofano, Aniello Riccio

**Affiliations:** Department of Engineering, University of Campania “Luigi Vanvitelli”, Via Roma 29, 81031 Aversa, Italy

**Keywords:** bird strike impact, lattice structures, multi-fidelity modeling, beam and solid finite elements, topology-dependent response, computational mechanics

## Abstract

Lattice-reinforced thin-walled structures offer a promising means of improving impact resistance while maintaining structural efficiency; however, their detailed numerical simulation can become computationally expensive under high-rate loading conditions. This study investigates how lattice topology influences the fidelity of reduced-order beam element models for bird strike impact analysis. Three lattice topologies, BCC, Diamond, and Kelvin, are evaluated using low-fidelity beam element and high-fidelity solid element models under identical geometry, material properties, boundary conditions, and impact loading at 68.4 m/s. The comparison considers transient and residual displacement, total and component-level energy absorption, stress distribution, and computational cost. The results demonstrate that modeling fidelity is strongly topology-dependent and response-dependent. For the sparse BCC architecture, low-fidelity predictions agree closely with the high-fidelity reference, with peak displacement and total energy discrepancies of approximately 10% and below 5%, respectively. In contrast, Kelvin and Diamond topologies exhibit larger discrepancies, particularly in residual deformation and component-level energy redistribution. Notably, the magnitude and character of the fidelity discrepancy vary with topology and response quantity, indicating that a universal correction factor based on a single response metric is inappropriate. Despite these limitations, beam element models reduce computational time by 74–86% relative to their solid element counterparts. The results provide quantitative guidance for selecting modeling fidelity according to lattice topology and structural response quantity within the investigated bird strike configuration.

## 1. Introduction

A bird strike is a critical impact loading condition for aircraft structures, particularly for leading-edge components that are directly exposed to high-velocity debris. Such events can induce severe local deformation and damage and may compromise the integrity of primary load-carrying components. Accurate prediction of the transient structural response is, therefore, an important requirement in the design of lightweight aerospace structures [1,2,3]. Conventional wing leading edges commonly employ thin-walled skins supported by rib–spar assemblies. Although these configurations provide established structural efficiency, simultaneously reducing mass and maintaining adequate impact resistance remains challenging [4,5].

Lattice-reinforced thin-walled structures provide an alternative approach in which stiffness, load paths, and deformation mechanisms can be tailored through lattice topology and geometric parameters [6,7,8]. The development of additive manufacturing has further increased the feasibility of complex cellular architectures that are difficult to manufacture using conventional processes. BCC, Diamond, and Kelvin lattices have consequently received considerable attention because their different connectivity and deformation characteristics can be exploited for lightweight and energy-absorbing structural applications [9,10]. However, most existing investigations of these architectures have focused on individual lattice specimens, simplified structural configurations, or quasi-static and moderate-rate loading. Their behavior when integrated within a geometrically representative thin-walled aerospace structure subjected to high-velocity bird strike loading remains less established.

A further challenge arises from the computational representation of the lattice itself. High-fidelity (HF) finite element models resolve the three-dimensional geometry of individual struts and their junctions using solid elements, allowing local deformation and stress redistribution to be represented explicitly. However, the resulting large number of elements and small stable time increments can make such models computationally expensive for explicit impact simulations. Low-fidelity (LF) beam element models offer a substantially reduced computational cost by representing the lattice struts as one-dimensional structural members. This simplification is attractive for parametric studies and design exploration, but it also modifies the representation of the junctions and their associated load transfer mechanisms [11,12]. Consequently, the important question is not whether beam models are generally accurate or inaccurate, but under which structural conditions and for which response quantities their predictions remain sufficiently consistent with a detailed solid representation. Moreover, recent theoretical developments have also emphasized the importance of nonlinear dynamic modeling for lightweight composite structures subjected to complex dynamic loading. Liu et al. [13] provided a recent comprehensive review of nonlinear dynamics, vibration behavior, and modeling approaches for lightweight composite structures, highlighting the importance of selecting appropriate modeling strategies for accurately capturing structural responses under dynamic conditions. These developments further motivate the use of controlled numerical modeling frameworks for lightweight lattice-reinforced aerospace structures subjected to high-rate impact loading.

Previous studies provide evidence that lattice junction representation is an important source of discrepancy between beam-based and detailed models. Cao et al. [14] showed that beam element predictions increasingly deviate from experimental behavior as strut dimensions change, owing in part to the inability of conventional beam formulations to represent the three-dimensional constraint at nodal intersections. Similarly, Liu et al. [15] showed that incorporating realistic nodal geometry significantly improves agreement between beam-based simulations and experiments, highlighting that junction modeling is a dominant source of fidelity error. Collectively, these studies indicate that nodal geometry is an important source of fidelity error in beam-based lattice modeling. However, most available evidence concerns individual lattice specimens and relatively simple loading conditions, leaving it unclear whether these observations remain valid when different lattice topologies are embedded within a thin-walled aerospace structure subjected to high-rate impact.

The computational motivation for using reduced-order models has also led to the development of multi-fidelity and surrogate-based approaches, in which inexpensive models are combined with a smaller number of high-fidelity evaluations [16,17]. The effectiveness of such approaches depends on the relationship between the low- and high-fidelity responses; strong and predictable correlation is required if the low-fidelity model is to provide useful information for design or optimization. For lattice structures, however, this relationship may not be uniform because topology changes connectivity, local constraint, stiffness distribution, and load transfer paths. More importantly, a model may reproduce one response quantity reasonably well while providing substantially different predictions for another quantity. Quantifying this topology- and response-dependent fidelity is, therefore, a prerequisite for the rational use of reduced-order lattice models in computationally intensive impact analyses.

Despite the progress in lattice mechanics and multi-fidelity modeling, a quantitative assessment of this issue for lattice-reinforced aerospace structures under bird strike impact remains limited. In particular, it is not sufficiently established how the discrepancy between beam- and solid element representations changes with lattice topology and response metric, or whether agreement in a global quantity such as total energy absorption necessarily implies agreement in residual deformation, component-level energy redistribution, or local stress fields. This distinction is important because BCC, Kelvin, and Diamond architectures possess different connectivity and deformation characteristics, which can alter the relative contribution of strut deformation and nodal constraint to the structural response. The resulting fidelity discrepancy may, therefore, vary substantially in magnitude and response-specific character, making a single topology-independent correction inappropriate.

Overall, the previous literature has established the effectiveness of beam-based lattice models for predicting global responses under quasi-static loading, while other investigations have focused on individual lattice topologies or isolated specimens. However, the applicability of such reduced-order representations to high-velocity bird strike loading within an integrated aerospace structure remains less established. The present study extends this body of work by comparing BCC, Diamond, and Kelvin architectures within the same UAV leading-edge configuration and by evaluating multiple response quantities, including transient and residual deformation, energy absorption, stress distribution, and computational cost.

Therefore, the novelty of the present work lies not in the use of beam and solid finite elements individually, nor in the general concept of multi-fidelity modeling, which are well-established approaches. Rather, the contribution is the systematic quantification of how the fidelity of beam-based lattice representations changes with both lattice topology and the structural response quantity of interest under bird strike loading. These configurations have identical loading, material, contact, and boundary conditions. The comparison extends beyond a single global response by considering transient and residual deformation, total and component-level energy absorption, stress distributions, and computational cost. This enables the identification of response quantities for which reduced-order beam models provide sufficiently close predictions and quantities for which three-dimensional solid modeling remains necessary. The resulting assessment, therefore, provides a response-dependent basis for selecting modeling fidelity rather than assuming a topology-independent equivalence between beam and solid representations.

## 2. Materials and Methodology

### 2.1. Wing Geometry Description

**Figure 1:**
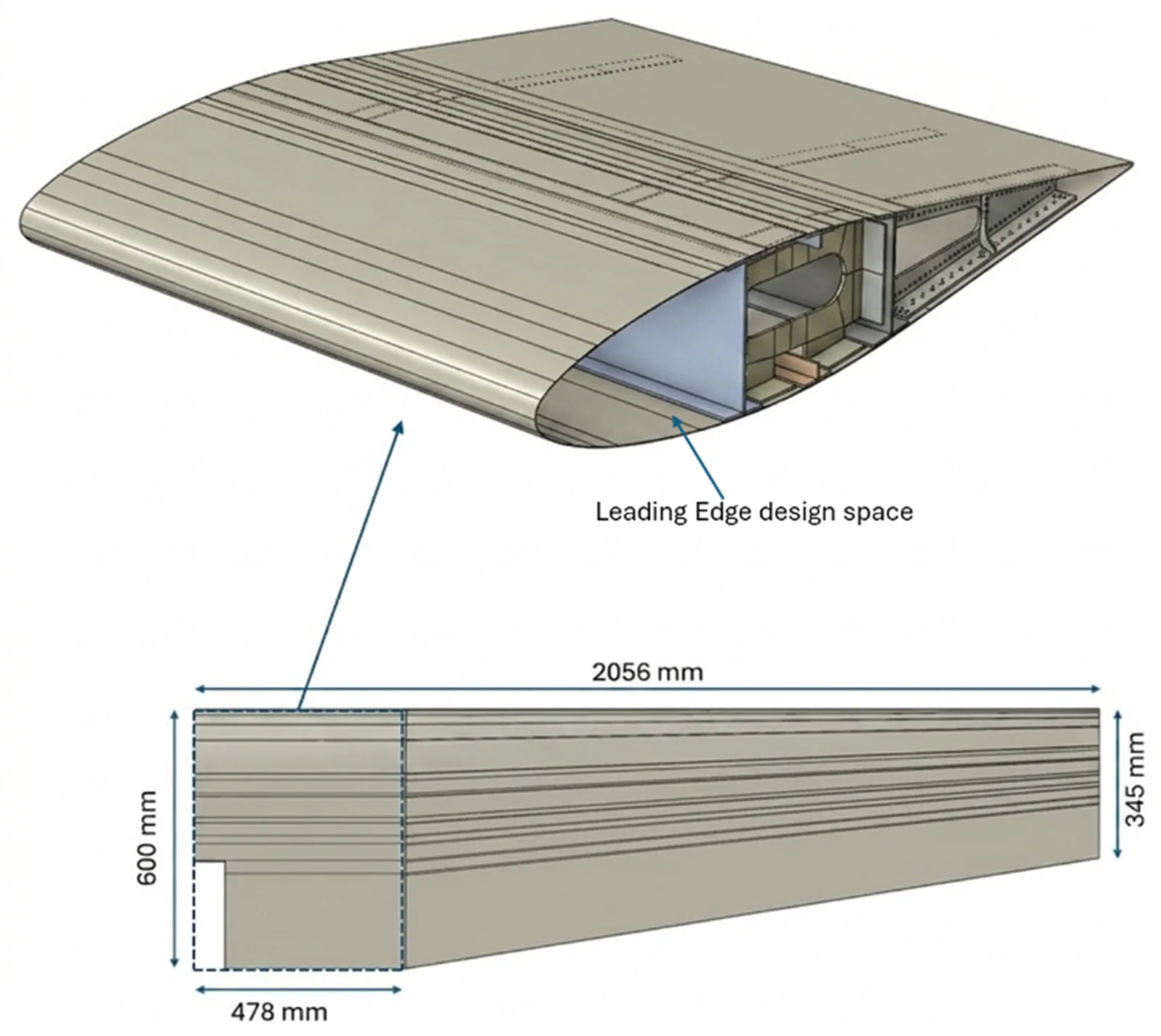
UAV wing and initial root section analyzed in current study.

A representative straight-section UAV wing leading edge was adopted as the structural configuration for the bird strike simulations. The configuration follows a CS-27-class UAV with a maximum take-off mass of 150 kg and a wing chord of 600 mm. The leading-edge structure consists of a thin CF/PA outer skin supported by an internal structural arrangement comprising a front spar and ribs. The baseline configuration and principal dimensions are shown in Figure 1.

The computational leading-edge domain has dimensions of approximately 478 mm × 190 mm × 103 mm in the longitudinal, spanwise, and thickness directions, respectively. The outer skin has a uniform thickness of 0.6 mm. In the lattice-reinforced configurations, the conventional internal arrangement is replaced by an architected lattice region while maintaining the same external leading-edge envelope. This provides a controlled structural configuration in which the influence of lattice topology and numerical modeling fidelity can be evaluated independently of changes in external geometry.

The CF/PA skin was retained because this material system has previously been employed within the research group for comparable lightweight UAV structural configurations [3,18]. The present study focuses on the response of the complete lattice-reinforced leading-edge system rather than on material-process characterization of the skin.

### 2.2. Lattice Architecture and Generation

**Figure 2:**
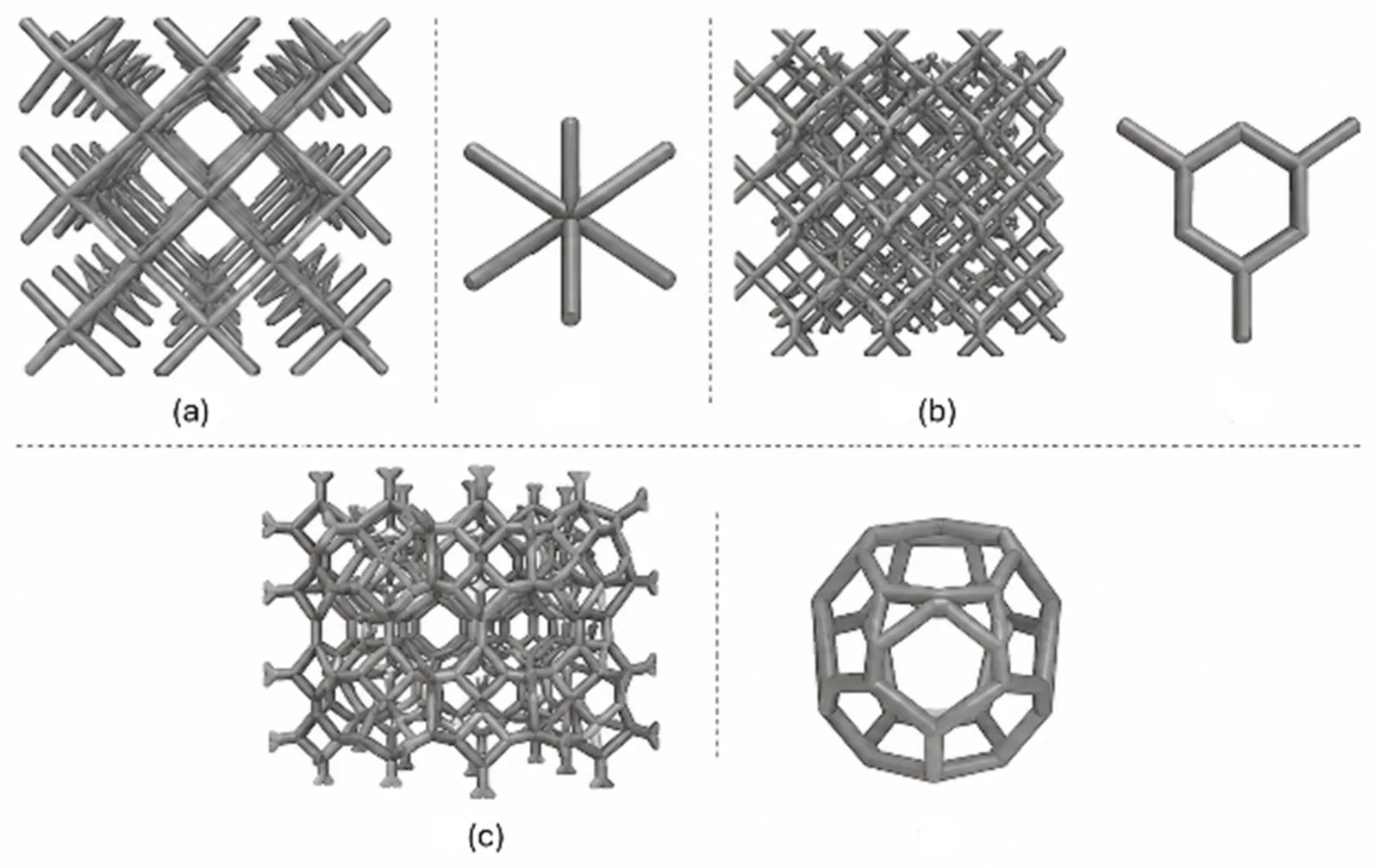
Unit cell topology: (**a**) BCC, (**b**) Diamond, and (**c**) Kelvin.

**Figure 3:**
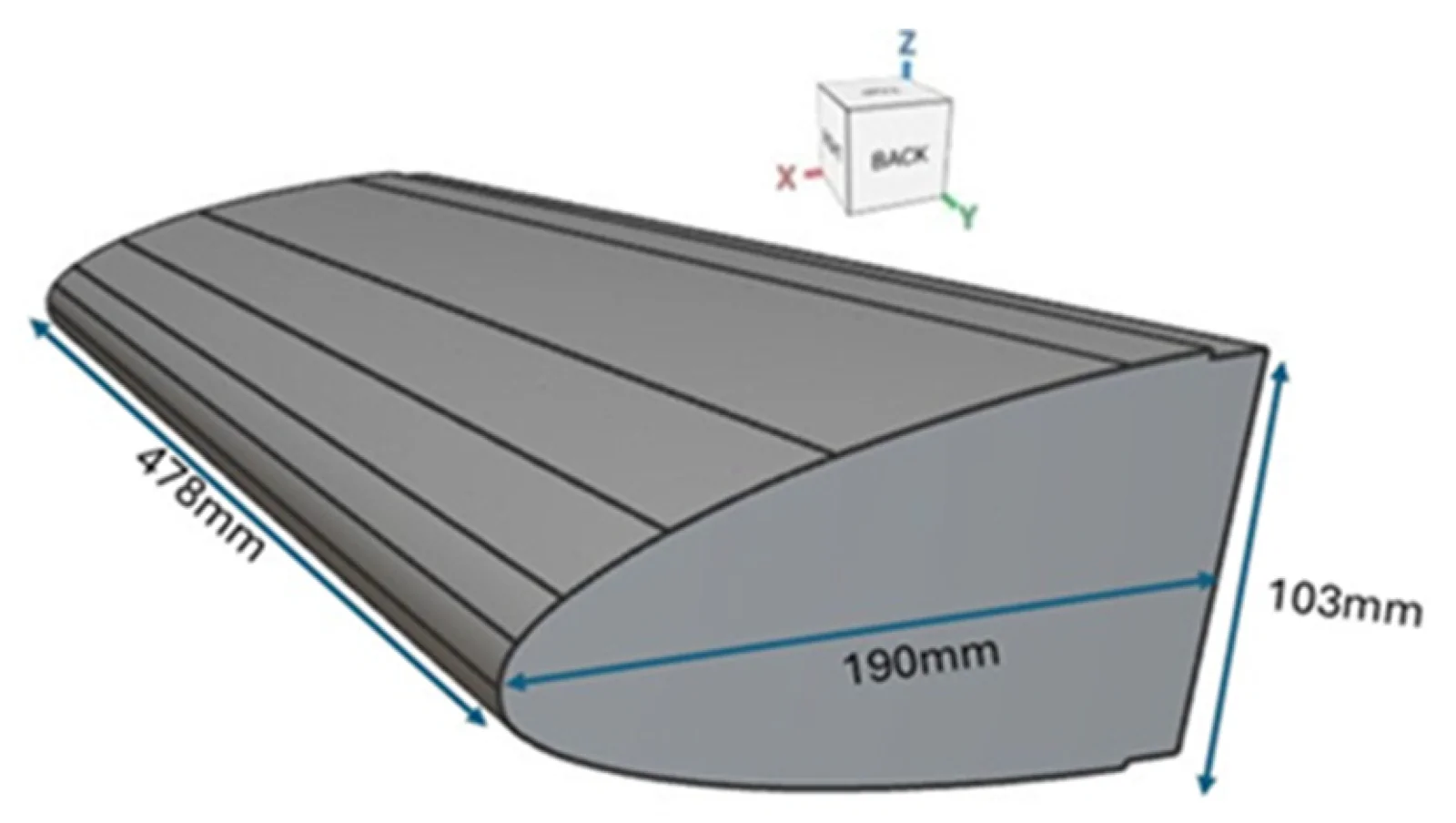
CAD solid geometry of L.E.

Three lattice topologies, body-centered cubic (BCC), Diamond, and Kelvin, were selected to provide architectures with distinct connectivity and load transfer characteristics. The selection was intended to provide a controlled comparison of modeling fidelity across topologies rather than to identify a single optimum lattice architecture. BCC provides a relatively sparse strut network with predominantly bending-dominated deformation, whereas Diamond and Kelvin provide more highly connected three-dimensional load paths. The three topologies are shown in Figure 2a–c. These differences are expected to influence the relative importance of strut deformation and nodal constraint and, consequently, the sensitivity of the structural response to the adopted finite element representation. The lattice architectures were generated parametrically using nTopology 5.22.2 and integrated within the leading-edge envelope Figure 3.

To ensure a controlled fidelity assessment, all lattice topologies were generated using an identical unit cell size of 50 mm × 50 mm × 50 mm and a constant strut diameter of 2 mm (d/L=0.04). The unit cell dimensions and strut diameter were deliberately kept constant for all three lattice topologies to establish a controlled basis for comparing topology-dependent modeling fidelity. This approach isolates the influence of lattice architecture and finite element representation without introducing additional variations associated with relative density or lattice scale. Previous studies have shown that relative density and strut dimensions can significantly influence the mechanical and energy absorption response of lattice structures under dynamic loading [19,20].

Accordingly, these parameters were controlled in the present study so that differences between the solid and beam element formulations could be assessed under consistent geometric conditions. A systematic parametric investigation of unit cell dimensions, strut diameter, and relative density is considered complementary to the present topology- and fidelity-focused investigation. These values were selected as a common baseline configuration to provide sufficient geometric resolution within the available leading-edge volume while maintaining computational tractability and geometric consistency across the investigated topologies. Therefore, the present study evaluates topology- and response-dependent modeling fidelity for this defined baseline lattice geometry rather than establishing fidelity trends over a range of relative densities or unit cell dimensions, as presented in Table 1.

Moreover, the leading-edge envelope has chordwise and thickness dimensions of 92.6 and 85 mm, respectively; the adopted 50 × 50 × 50 mm unit cell results in only one to two cells across these dimensions. The lattice was, therefore, truncated by the leading-edge boundaries, with intersecting struts terminated at the corresponding skin/interface surfaces. The same truncated geometry and boundary configuration were used in both HF and LF models. Although boundary truncation may influence the absolute structural response, its effect was kept consistent between the two fidelity levels.

For the high-fidelity (HF) representation, the complete three-dimensional lattice geometry, including the struts and nodal junctions, was discretized using solid elements. For the low-fidelity (LF) representation, the lattice struts were represented using beam elements while retaining the same global lattice geometry and connectivity. Thus, the two models differed primarily in their representation of the lattice mechanics rather than in the overall structural configuration.

**Figure 4:**
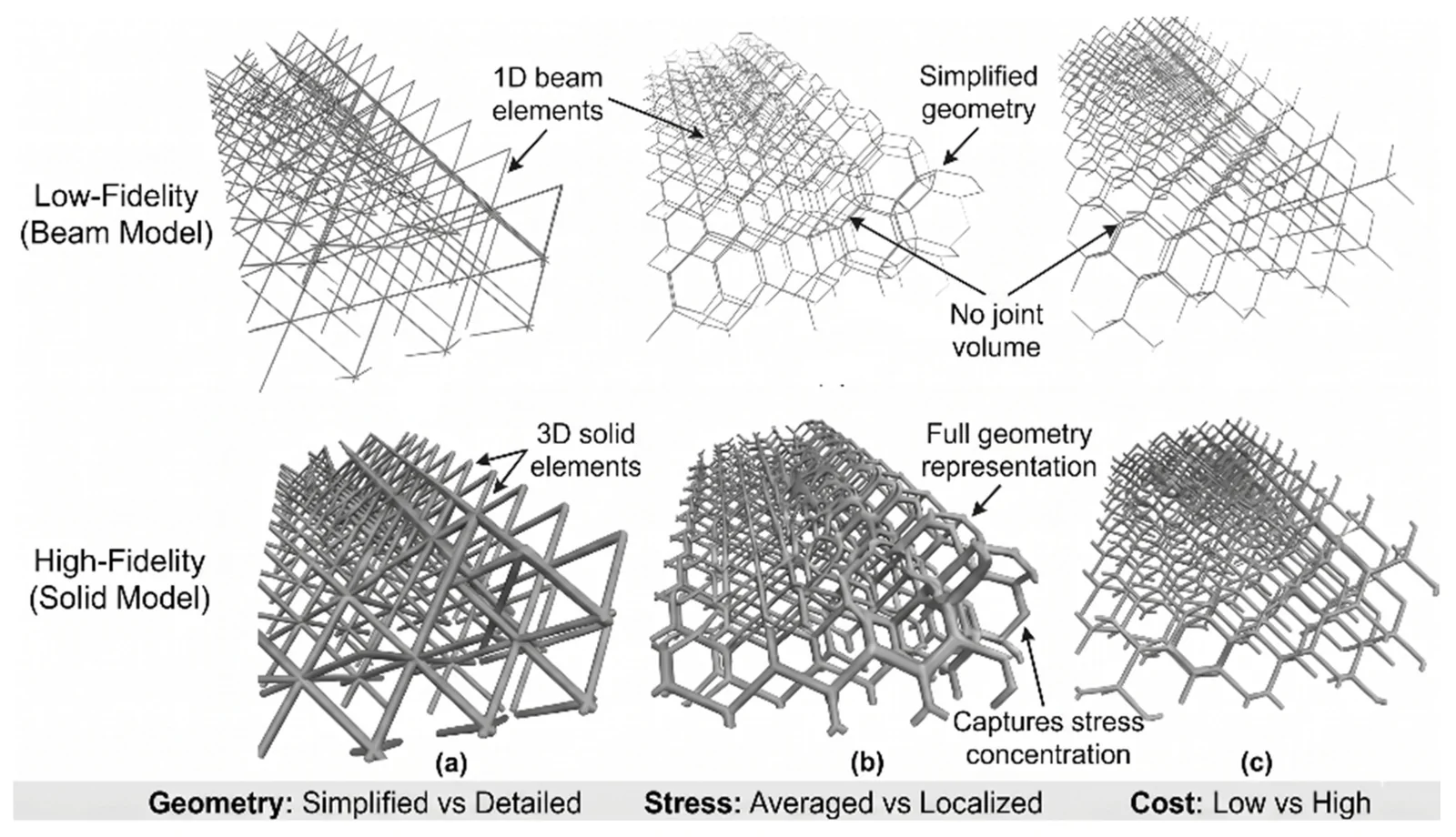
High-fidelity (solid element) and low-fidelity (beam element) representations of the investigated lattice topologies: (**a**) BCC, (**b**) Kelvin, and (**c**) Diamond. Both fidelity levels were generated from identical CAD geometry, differing only in numerical discretization.

The resulting lattice-infilled L.E models are shown in Figure 4 for all three topologies in both their solid and beam representations. The two fidelity levels exhibit substantially different numerical representations. The solid models fully resolve the three-dimensional geometry of each strut, nodal junction, and surface, whereas the beam models reduce each strut to a line element.

To ensure an unbiased fidelity assessment, all comparisons between the high-fidelity and low-fidelity models were performed using identical material properties, boundary conditions, contact definitions, impact conditions, lattice topology, unit cell dimensions, and strut diameter. Consequently, the finite element representation of the lattice remained the principal modeling variable between the two approaches. Moreover, this controlled baseline configuration was adopted specifically to isolate the effects of lattice topology and numerical discretization; consequently, the present fidelity trends should not be interpreted as independent of relative density, strut diameter, or unit cell dimensions.

### 2.3. Numerical Modeling Framework

**Figure 5:**
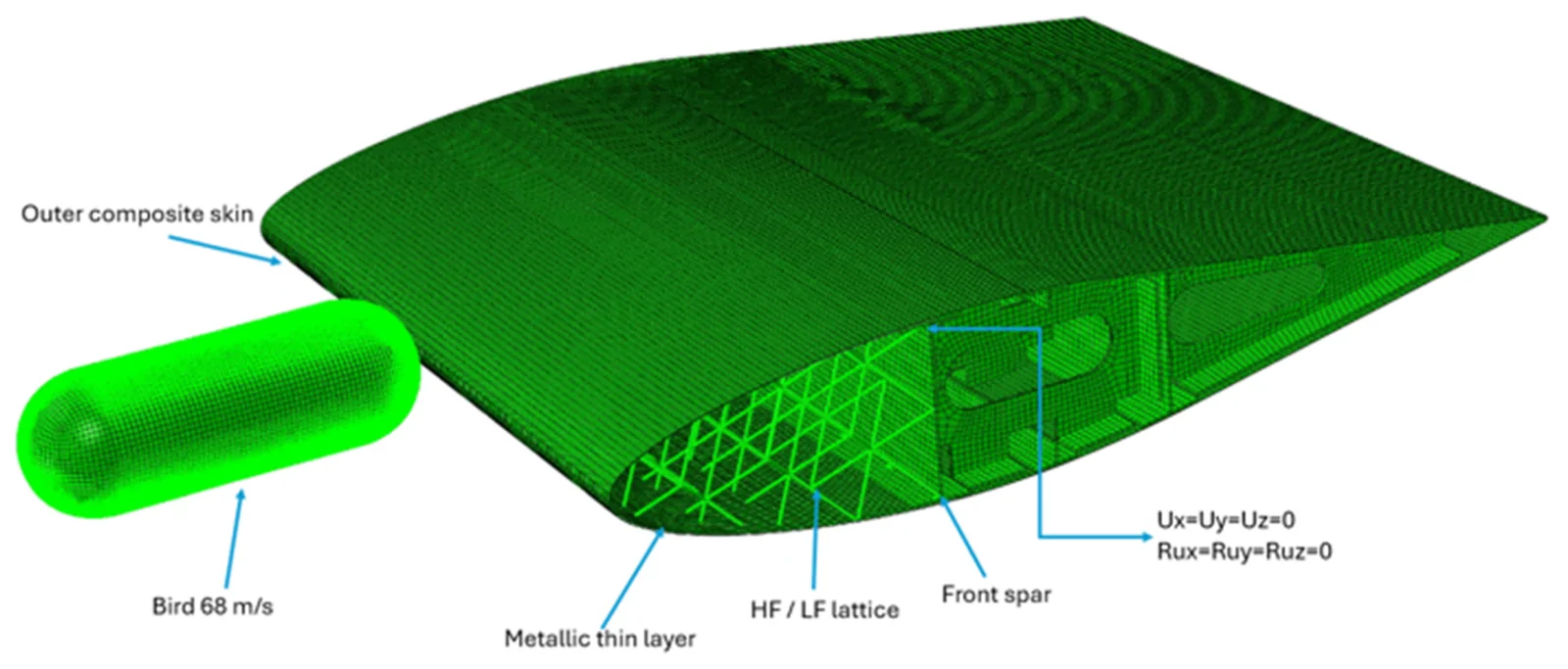
Numerical model of comparative analysis in Abaqus.

A consistent finite element framework was developed in Abaqus/Explicit 2020 to investigate the bird strike response of lattice-reinforced UAV leading-edge structures using both high-fidelity (HF) and low-fidelity (LF) models. To ensure a controlled assessment of modeling fidelity, all simulations employed identical external geometry, lattice topology, unit cell dimensions, material properties, impact conditions, boundary conditions, and contact definitions, while the finite element representation of the lattice remained the principal modeling difference between the two approaches. As illustrated in Figure 5, the numerical model consists of an outer composite skin, an internal lattice-reinforced leading-edge region, a front spar, ribs, and a 0.1 mm metallic interface layer positioned between the lattice and composite skin to facilitate efficient load transfer. The lattice domain was represented using either three-dimensional solid elements (HF) or one-dimensional B31 beam elements (LF), while maintaining identical geometric characteristics to ensure that differences in the predicted structural response arise solely from the modeling fidelity.

A soft-body bird projectile was modeled using the Smoothed Particle Hydrodynamics (SPH) approach and assigned an initial impact velocity of 68.4 m/s, consistent with the previous bird strike studies of the same class [18]. The bird was represented as a cylindrical gelatin projectile with a length of 224 mm, a diameter equal to half of its length, and a density of 950 kg/m^3^. A Us-Up equation of state was adopted to describe its hydrodynamic response during impact. The bird model was discretized using 46,560 C3D8R elements, which were subsequently converted into SPH particles in Abaqus/Explicit. The bird model was previously validated with experimental work of Lavoie et al. [21] in our previous study [3,22]. Additionally, the bird density of 950 kg/m^3^ and the (Us-Up) equation-of-state formulation are also consistent with independent bird strike studies employing Abaqus/Explicit and SPH formulations for aerospace impact problems [23,24]. These studies report comparable bird densities and hydrodynamic EOS formulations, supporting the use of the adopted approach for representing the soft-body response of the bird. The influence of SPH particle resolution and EOS parameters was not independently varied in the present study; therefore, the present work does not claim sensitivity-independent predictions with respect to these parameters. The validated formulation is, therefore, adopted here without further calibration.

Boundary conditions were applied at the rear section of the leading edge by constraining all translational and rotational degrees of freedom to represent the wing root attachment. Furthermore, previous studies have shown that lattice response can be influenced by the number of unit cells and the applied boundary conditions [25]. In the present study, the lattices were integrated throughout the leading-edge domain and coupled with the surrounding structural components, with identical boundary conditions applied to all topologies and fidelity levels. Thus, these effects were kept consistent in the HF-LF comparison, although their potential influence on the absolute response is acknowledged.

General contact with hard normal contact and frictionless tangential behavior was defined for all interacting surfaces, while bonded interfaces between structural components were modeled using tie constraints. A dynamic explicit analysis was performed with a total simulation time of 0.01 s. A uniform target time increment of $5\times 10^{-7}$ s was introduced through mass scaling at the beginning of the analysis to improve computational efficiency. To assess the influence of the adopted mass-scaling treatment, an additional simulation was performed without mass scaling using otherwise identical model, material, contact, boundary condition, and loading definitions. The maximum structural displacement was approximately 32.8 mm for the mass-scaled model and 34.5 mm for the corresponding simulation without mass scaling, resulting in a difference of approximately 5%. The deformation patterns were also qualitatively consistent between the two cases. The internal energy histories exhibited some difference in absolute magnitude, indicating that mass scaling has some influence on the energy response. Therefore, the component-level energy quantities reported in this study should be interpreted primarily for comparative HF–LF assessment under the consistently applied numerical framework rather than as mass-scaling-independent absolute energy predictions.

In addition to that, recent studies have similarly highlighted the sensitivity of explicit high-rate simulations to constitutive parameters and numerical modeling choices. In particular, Beecher et al. [26] reported significant sensitivity of Johnson–Cook predictions to the strain rate parameter (C) and thermal-softening exponent (m), while Tuninetti et al. [27] demonstrated the importance of mesh sensitivity and constitutive-model validity under extreme thermo-mechanical loading. These findings emphasize the importance of considering numerical and constitutive uncertainty when interpreting quantitative impact predictions. The present study does not perform a systematic uncertainty quantification analysis; instead, the additional mass-scaling verification provides a reproducibility check for the principal structural response used in the modeling-fidelity comparison.

All simulations were performed on an HP Z8 G4 workstation equipped with dual Intel^®^ Xeon^®^ Gold 6258R processors (56 physical cores), 512 GB RAM, and an NVIDIA Quadro GV100 GPU with 32 GB memory, and parallel processing was enabled throughout the analyses.

### 2.4. Constitutive Material Models

The structural components, including the front spar, ribs, metallic interface layer, and lattice structures, were modeled using aluminum alloy Al2024-T3. The elastic-plastic behavior of the material was described using the Johnson–Cook constitutive model, which was selected because it represents strain hardening and strain rate sensitivity under high-velocity impact loading and has been widely used for Al2024-T3. Ductile damage initiation and displacement-based damage evolution were incorporated for the Al2024-T3 components and the CF/PA skin to represent progressive material degradation under impact loading. Element deletion was monitored through the STATUS variable to assess whether complete material removal occurred during the impact event. The material properties adopted in this study were taken from the validated experimental studies of Vershinin [28] and Lesuer [29], and they are summarized in Table 2. Moreover, the Johnson–Cook parameters were kept identical between the HF and LF formulations so that differences in the predicted response could be assessed without introducing constitutive model variability.

The outer skin was modeled using a 0.6 mm thick carbon-fiber-reinforced polyamide (CF/PA) composite. The material corresponds to an additively manufactured short-carbon-fiber-reinforced thermoplastic rather than a conventional continuous-fiber UD or woven laminate. Therefore, a ply-based orthotropic or transversely isotropic formulation was not adopted. Instead, an equivalent homogeneous material representation was employed using the effective properties of the CF/PA material. The material formulation and properties were adopted from the validated material model used in our previous studies [18,30]. This approach provides a consistent representation of the macroscopic response of the additively manufactured short-fiber composite while avoiding the introduction of fiber-orientation and ply-level parameters that are not defined for the material considered here in Table 3.

The outer skin was modeled using a 0.6 mm thick carbon-fiber-reinforced polyamide (CF/PA) composite, which has been widely adopted for lightweight UAV structures owing to its favorable stiffness-to-weight ratio and impact resistance. Although the outer skin was represented as a homogeneous material rather than a laminated composite, ductile damage initiation and displacement-based damage evolution were incorporated to reproduce progressive material degradation under impact loading.

### 2.5. Mesh Convergence and Discretization

To ensure numerical reliability, mesh convergence studies were conducted for both HF (solid) and LF (beam) lattice models. The BCC configuration was selected as the representative case for establishing the discretization requirements because the objective of this convergence assessment was to determine the element resolution required for the common strut geometry used throughout the study. The resulting solid element size and beam subdivision level were then applied consistently to the Diamond and Kelvin configurations, which were generated using the same 2 mm strut diameter and the same geometric modeling procedure. This ensured a consistent discretization criterion across the three topologies and avoided introducing topology-dependent mesh parameters into the HF-LF comparison. Mesh convergence was assessed using the absorbed energy response of the lattice. For the solid element model, element sizes of 0.8, 1.0, 1.2, 1.4, and 1.6 mm were investigated.

**Figure 6:**
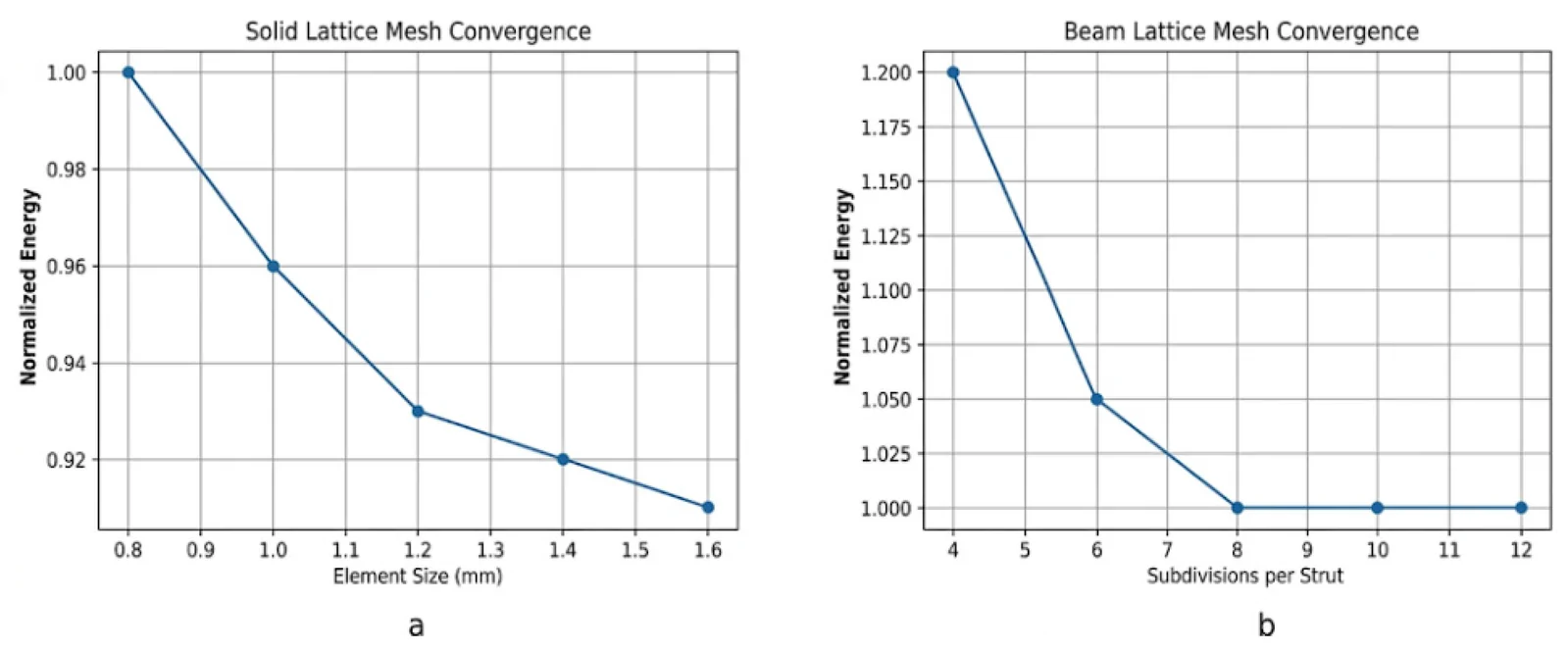
Mesh convergence w.r.t lattice energy absorption: (**a**) solid elements and (**b**) beam elements.

The corresponding absorbed energies were 615.913, 599.170, 582.430, 581.880, and 576.358 J, respectively. The absorbed energy was normalized according to $E_{norm}\left(h\right)=E\left(h\right)/E\left(0.8\;mm\right)$, where the 0.8 mm mesh represents the finest discretisation and was used as the reference. The resulting normalized values were 1.000, 0.972, 0.946, 0.945, and 0.936, respectively. Although the 1.2 mm mesh differs from the finest mesh by approximately 5.1%, the change between the 1.2 and 1.4 mm meshes is less than 0.1%, while the difference between the 1.2 and 1.6 mm meshes is approximately 1.0%. Thus, the absorbed energy response shows limited sensitivity to further mesh coarsening beyond 1.2 mm, and 1.2 mm was selected as a practical compromise between numerical resolution and computational cost. For the beam formulation, convergence was evaluated using 4, 6, 8, 10, and 12 subdivisions per strut. The energy histories show that the responses for 8, 10, and 12 subdivisions are closely grouped, whereas larger differences are observed for 4 and 6 subdivisions. Accordingly, 8 subdivisions per strut were adopted for the subsequent beam element simulations. The energy values are normalized with respect to the converged solution to facilitate comparison of mesh sensitivity and highlight convergence trends as presented in Figure 6.

Based on these results, all subsequent simulations employ a discretization of 1.2 mm for solid lattices and 8 subdivisions per strut for beam lattices. These discretization parameters were subsequently applied consistently to all three lattice topologies, providing a common numerical basis for the multi-fidelity comparison. This ensures mesh-independent predictions while maintaining computational efficiency for the multi-fidelity comparison. The following tables (Table 4 and Table 5) present the total number of nodes and elements for the entire structure as well as for the lattice topologies, in order to illustrate the complexity of both models.

## 3. Results and Discussion

### 3.1. Deformation Response

**Figure 7:**
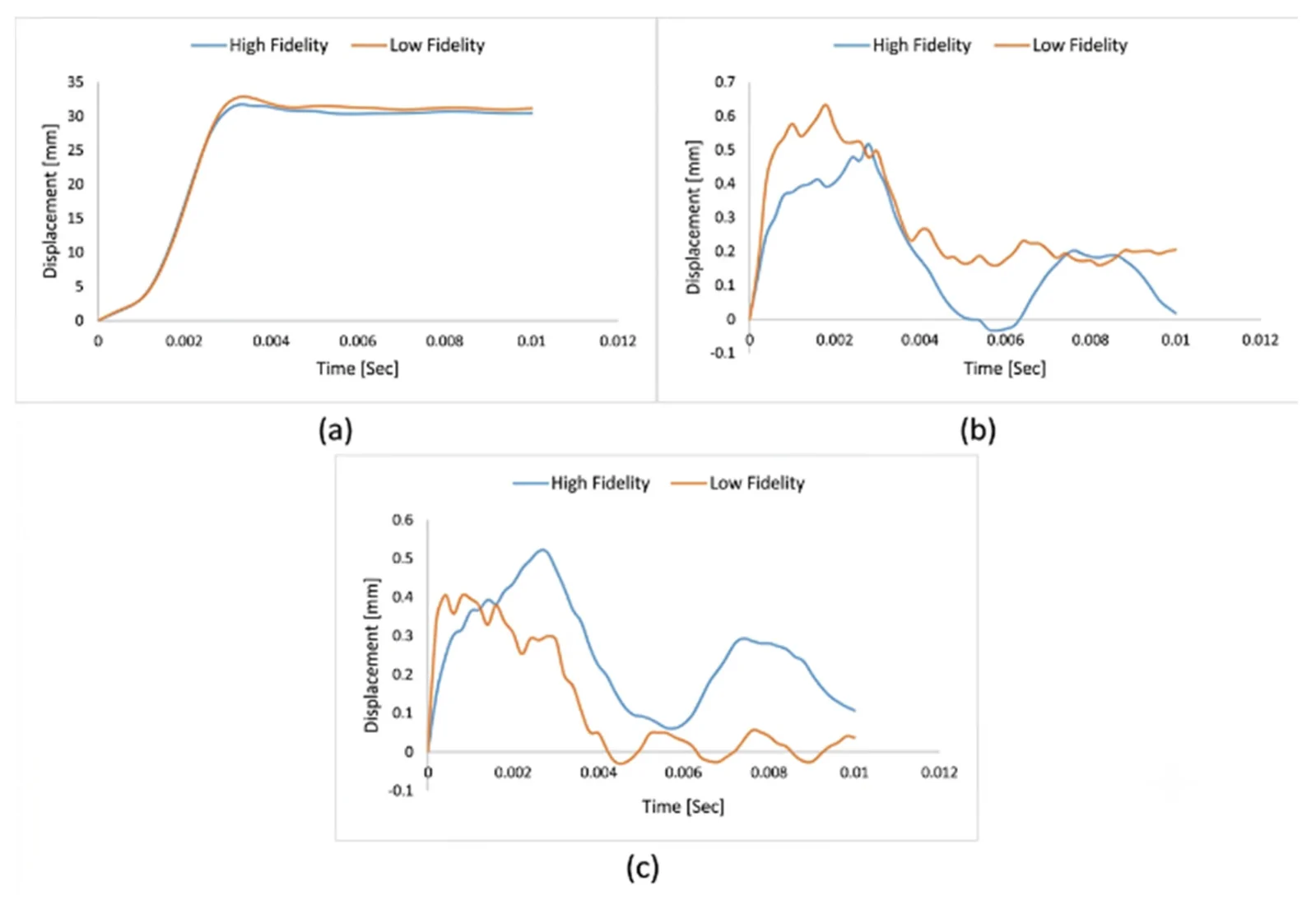
Time histories of the central node displacement for the (**a**) BCC, (**b**) Kelvin, and (**c**) Diamond lattice topologies predicted using high-fidelity (HF) and low-fidelity (LF) models.

Figure 7 compares the transient and residual displacement responses predicted by the HF solid element and LF beam element models for the BCC, Kelvin, and Diamond lattice configurations. Although the two formulations use identical geometry, material properties, impact conditions, and boundary conditions, their agreement varies systematically with lattice topology. This indicates that the influence of modeling fidelity is not uniform across the investigated architectures.

For the BCC configuration, the LF model closely reproduces the global deformation response of the HF model. The peak displacement predicted by the LF formulation differs from the HF result by approximately 10%, while the residual displacement remains within a few percent of the reference value. The relatively small discrepancy is consistent with the predominantly strut-level deformation and comparatively sparse connectivity of the BCC architecture, for which the beam formulation can represent the dominant load-carrying behavior reasonably well.

A substantially different response is obtained for the Kelvin lattice. The LF model predicts a higher peak displacement than the HF model, corresponding to an error of approximately 40%. The difference becomes more pronounced in the residual response, where the LF formulation substantially overpredicts the permanent deformation. The discrepancy indicates that the simplified beam representation does not reproduce the stiffness and load transfer constraints associated with the highly connected lattice junctions with the same fidelity as the three-dimensional solid representation.

**Figure 8:**
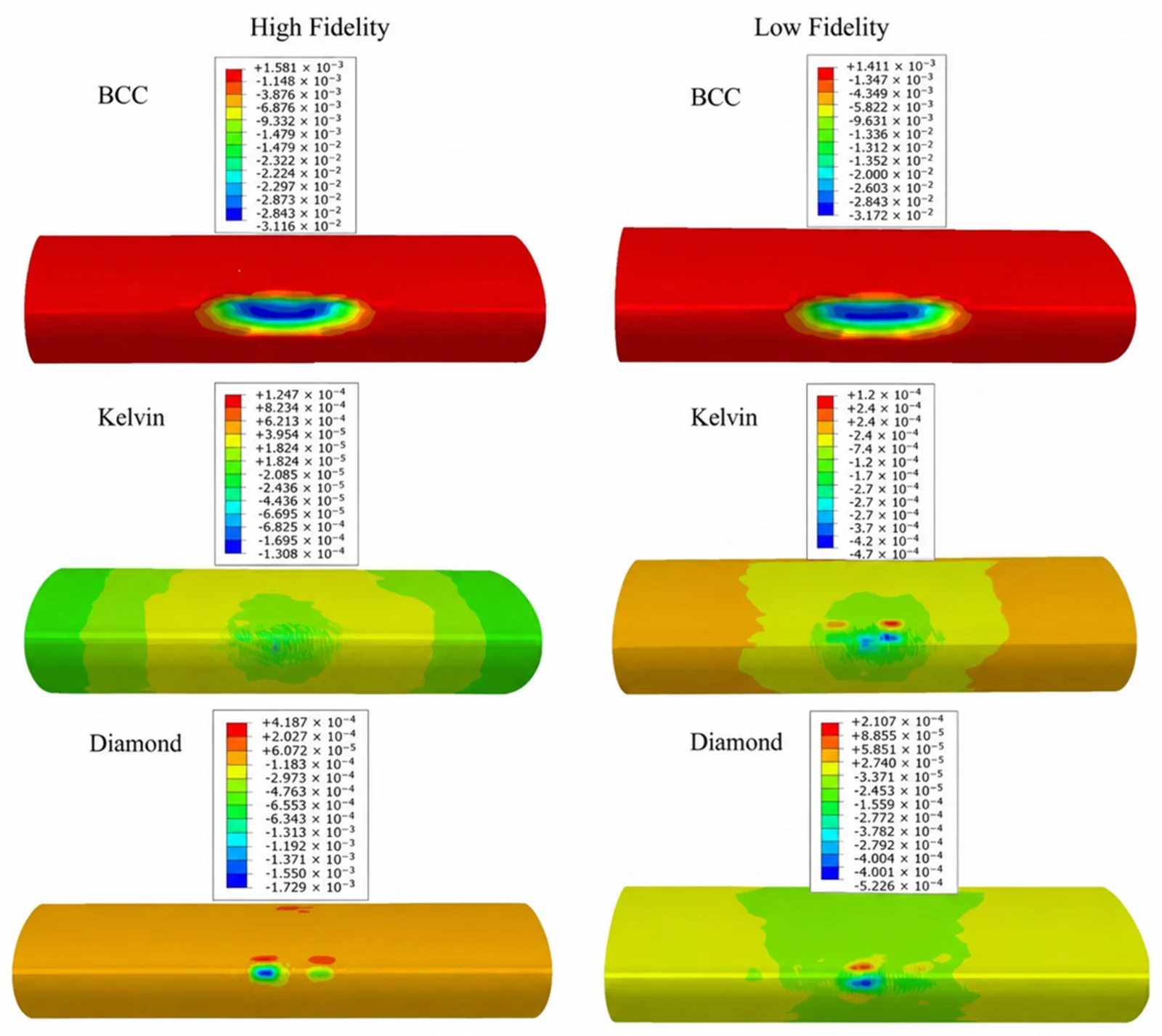
Comparison of residual U1 displacement contours predicted by the high-fidelity (HF) and low-fidelity (LF) models for the BCC, Kelvin, and Diamond lattice topologies.

The Diamond lattice exhibits an intermediate discrepancy in peak displacement, with an LF error of approximately 18%, but a considerably larger difference in the residual response. This behavior further demonstrates that peak displacement alone does not provide a sufficient measure of modeling fidelity. Two models may exhibit relatively similar transient global deformation while predicting substantially different post-impact structural states, as presented in Figure 8.

The comparison, therefore, reveals an important feature of the lattice response: modeling fidelity is both topology-dependent and response-dependent. The BCC architecture exhibits relatively stable agreement between the HF and LF formulations for the global deformation response, whereas the more highly connected Kelvin and Diamond architectures show greater sensitivity, particularly in residual deformation. This behavior is attributed to the increasing importance of three-dimensional load transfer and nodal constraint, which cannot be represented explicitly by a one-dimensional beam formulation. Accordingly, the suitability of an LF model should be assessed against the specific structural response required from the analysis rather than against a single global displacement metric.

**Figure 9:**
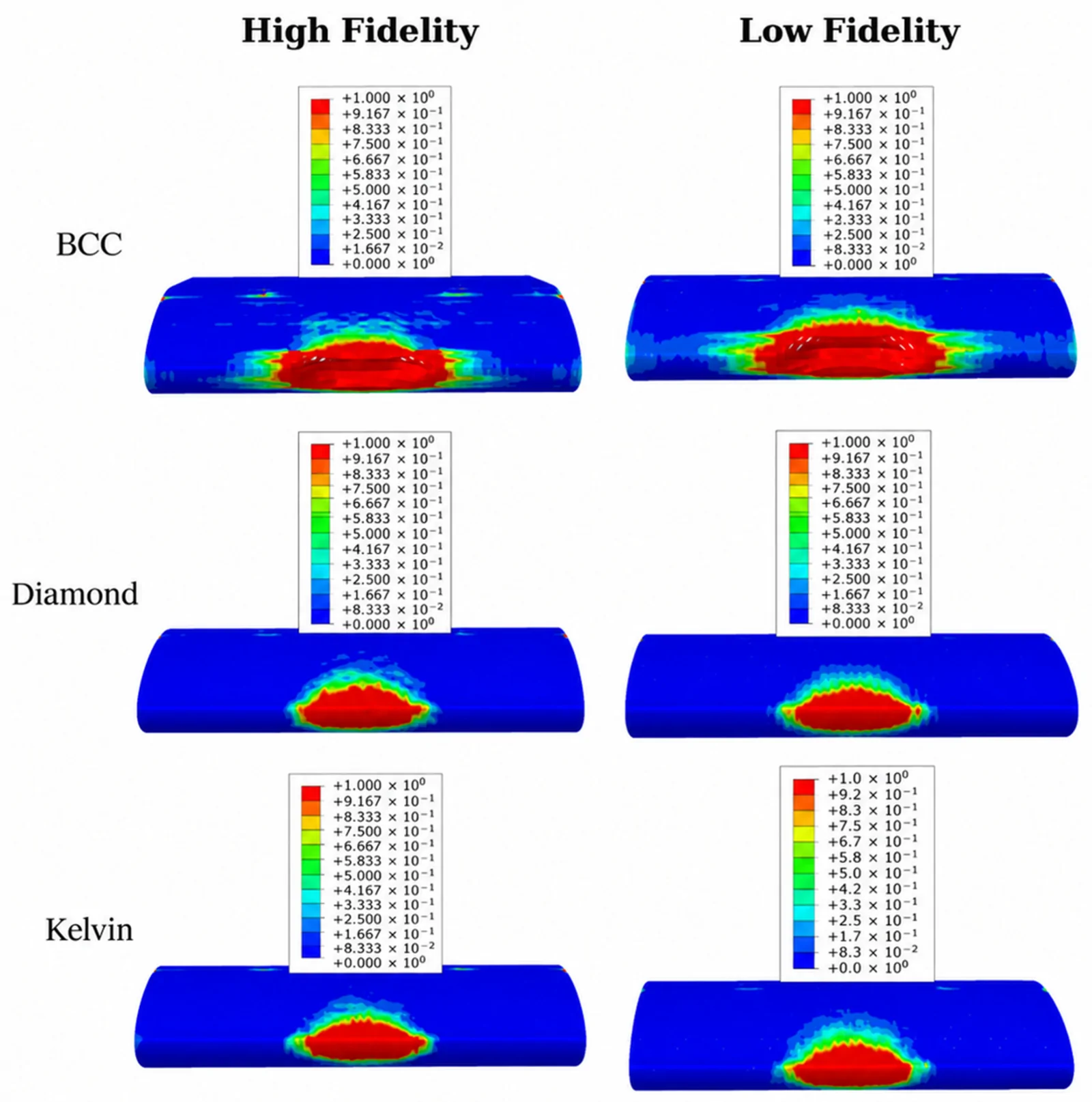
Ductile damage initiation criterion (DUCRT) for the investigated lattice-reinforced leading-edge models: BCC, Diamond, and Kelvin for high-fidelity and low-fidelity representations.

The local damage response was further examined using the ductile damage initiation criterion (DUCRT). As shown in Figure 9, DUCRT reaches its limiting value of 1 in the region directly affected by the bird impact for all three lattice topologies and for both HF and LF models, indicating local attainment of the ductile damage initiation criterion. The damage initiation region remains concentrated around the impact location, although its spatial distribution varies with lattice topology and modeling fidelity. The corresponding STATUS output remained equal to 1 for the investigated structural elements, indicating that element deletion was not observed during the simulated impact event. Therefore, although localized plastic deformation and ductile damage initiation occur in the impact region, the simulations do not indicate complete element removal associated with strut fracture or skin perforation. The damage response is, therefore, interpreted primarily as localized damage initiation and progressive deformation rather than complete structural failure.

### 3.2. Energy Absorption and Energy Partitioning

**Figure 10:**
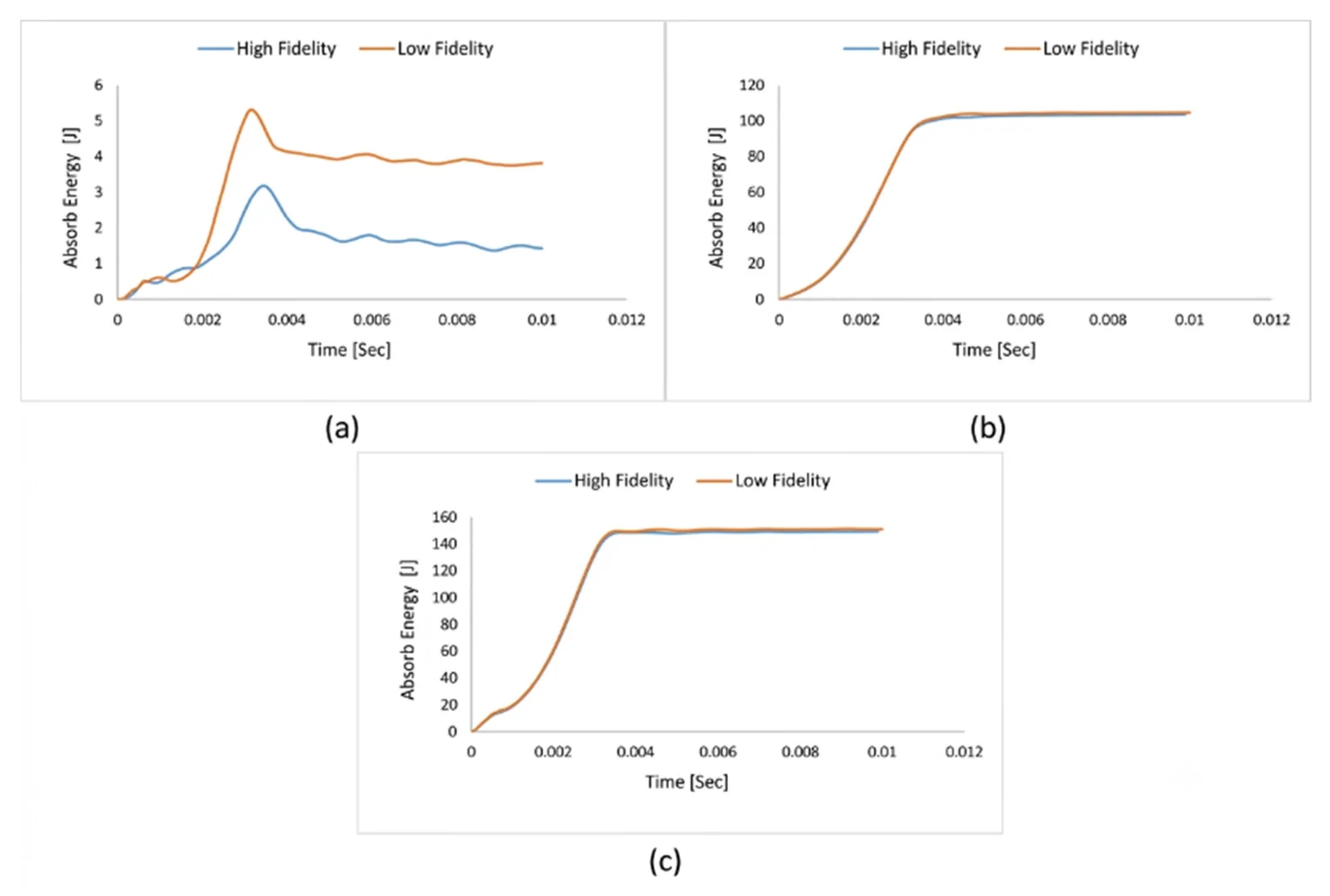
Comparison of absorbed energy histories predicted by the HF and LF models for the BCC lattice: (**a**) lattice structure, (**b**) outer composite skin, and (**c**) complete leading-edge structure.

**Figure 11:**
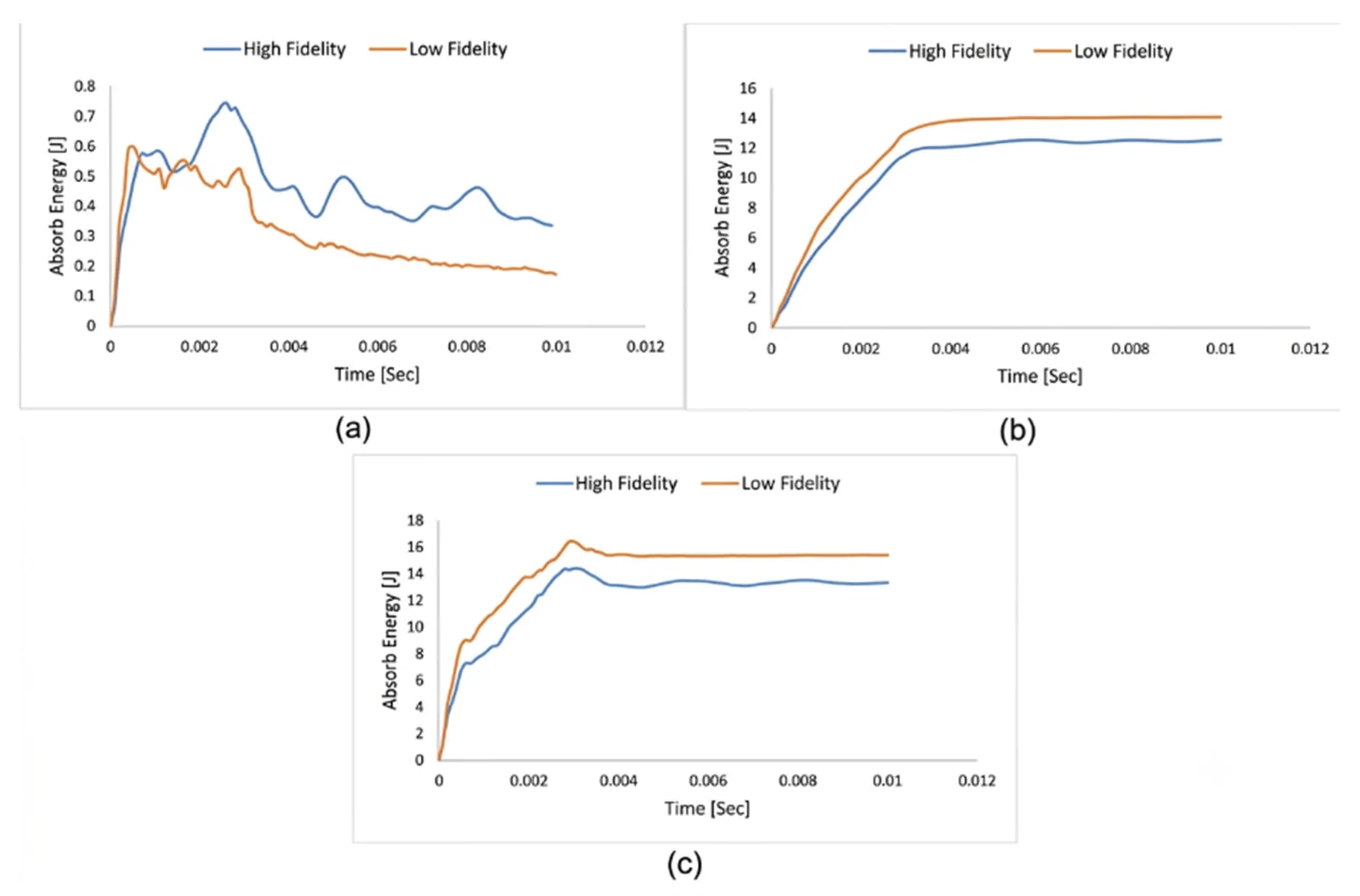
Comparison of absorbed energy histories predicted by the HF and LF models for the Kelvin lattice: (**a**) lattice structure, (**b**) outer composite skin, and (**c**) complete leading-edge structure.

**Figure 12:**
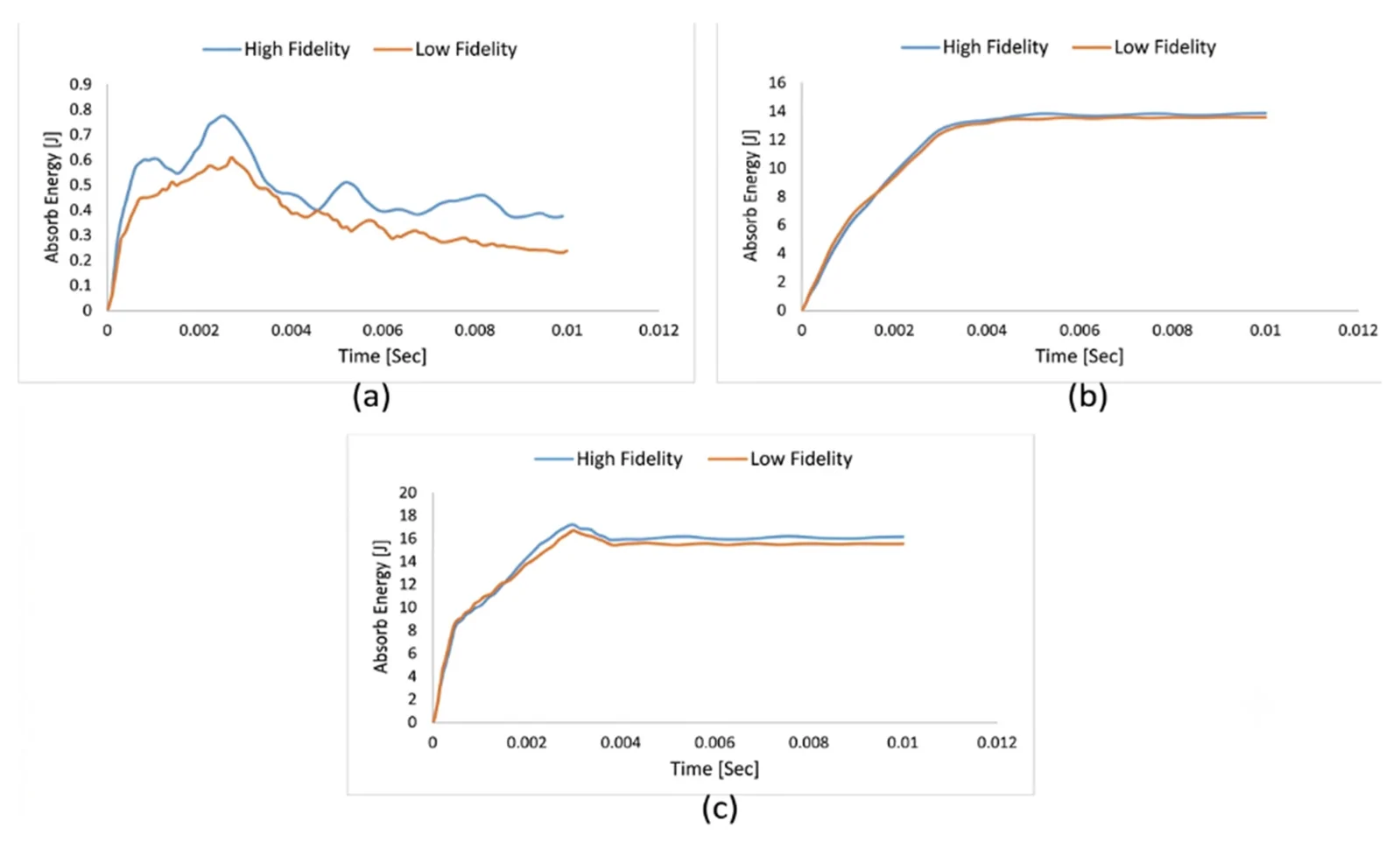
Comparison of absorbed energy histories predicted by the HF and LF models for the Diamond lattice: (**a**) lattice structure, (**b**) outer composite skin, and (**c**) complete leading-edge structure.

Energy absorption is a key indicator of impact resistance because it describes how the kinetic energy of the bird is dissipated among the lattice, the composite skin, and the overall leading-edge structure. To evaluate the influence of modeling fidelity on energy transfer mechanisms, the absorbed energy histories of the lattice, outer composite skin, and complete structure were compared for the three investigated lattice topologies (Figure 10, Figure 11 and Figure 12).

For the BCC lattice, the LF model overpredicts the energy absorbed by the lattice while the energy absorbed by the composite skin remains nearly identical to that predicted by the HF model (Figure 10). Consequently, the total structural energy differs by less than 5%, indicating that the simplified beam representation adequately reproduces the global energy dissipation of this sparse, bending-dominated topology. Although the internal energy partitioning differs slightly between the two fidelity levels, these differences have little influence on the overall structural response because the composite skin remains the primary energy-absorbing component.

A different behavior is observed for the Kelvin and Diamond lattices (Figure 11 and Figure 12), where the energy distribution becomes increasingly sensitive to modeling fidelity. For both topologies, the LF model underpredicts the energy absorbed by the lattice while transferring a larger proportion of the impact energy to the composite skin. This redistribution is most pronounced for the Kelvin lattice, whereas the Diamond lattice exhibits smaller differences despite a similar trend. Nevertheless, the total absorbed energy remains within approximately 15% for Kelvin and 5% for Diamond, demonstrating that good agreement in global energy does not necessarily imply accurate prediction of the underlying energy transfer mechanisms.

The observed differences originate from the fundamentally different representations of the lattice architecture in the two modeling approaches. In the HF models, the solid element discretization captures the three-dimensional stiffness of lattice junctions and the local interactions between intersecting struts, allowing realistic stress redistribution and energy dissipation within the lattice. In contrast, the LF beam representation simplifies each strut as a one-dimensional structural member and cannot accurately reproduce the stiffness contribution of complex nodal regions. As a result, part of the impact energy that should be dissipated within the lattice is instead transferred to the surrounding composite skin, leading to the altered energy partitioning observed for the Kelvin and Diamond topologies.

Moreover, the opposite signs of the lattice energy discrepancy observed for BCC compared with Kelvin and Diamond can be attributed to the interaction between lattice topology and the kinematic idealization of the beam representation. In the solid element models, the finite volume strut junctions can develop three-dimensional, multi-axial deformation and local constraint, whereas the B31 representation idealizes the same connections through one-dimensional beam kinematics. For the BCC topology, this idealization permits a different distribution of axial and bending deformation along the struts, resulting in a higher amount of work being attributed to the lattice in the LF model. In contrast, for the more highly connected Kelvin and Diamond architectures, local constraint and load transfer behavior at interconnected junctions become more influential. The beam representation cannot fully reproduce the three-dimensional junction response resolved by the solid model, leading to lower predicted lattice energy absorption. Thus, the reversal in the sign of the component-level energy discrepancy should not be attributed solely to the conventional bending- versus stretching-dominated classification but rather to the interaction between topology, junction constraint, and the kinematic assumptions of the beam idealization. Importantly, these component-level differences are accompanied by a redistribution of absorbed energy between the lattice and surrounding skin, which explains why the discrepancy in total structural energy remains substantially smaller than the discrepancy in individual components.

The energy results, therefore, reinforce the topology- and response-dependent nature of modeling fidelity identified from the deformation analysis. For preliminary design studies in which only total energy absorption is required, the LF formulation can provide substantial computational savings. However, when the objective is to understand energy redistribution between structural components or to evaluate topology-specific load transfer behavior, the HF representation provides a more reliable description.

### 3.3. Stress Distribution and Load Transfer Mechanisms

Stress distribution provides important insight into the load transfer mechanisms and potential failure locations within lattice-reinforced structures. Unlike displacement or energy absorption, a direct quantitative comparison of stress magnitudes between the HF solid element and LF beam element models is not physically meaningful because the two modeling approaches employ different stress formulations and dimensional representations. Consequently, the present analysis focuses on the qualitative comparison of stress distribution patterns to evaluate how modeling fidelity influences the prediction of load paths and stress concentrations. Since the outer skin consists of randomly distributed short carbon fibers embedded within a polyamide matrix, it was modeled as an equivalent homogeneous material. Accordingly, the equivalent homogeneous CF/PA representation was evaluated using the von Mises stress field as a consistent comparative measure of the global stress distribution between the investigated configurations.

**Figure 13:**
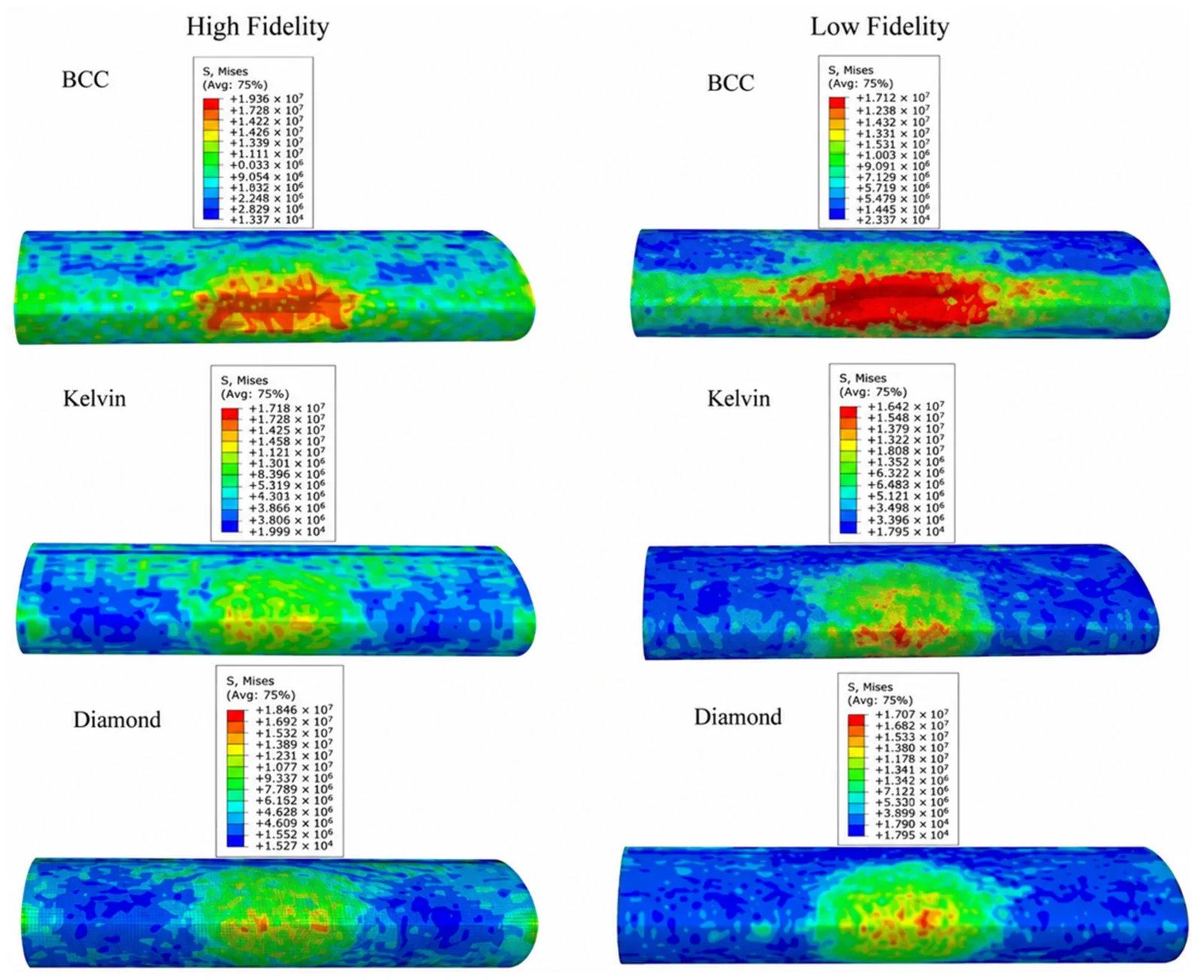
Comparison of von Mises (Pa) stress distributions in the composite skin predicted by the HF and LF models for the BCC, Kelvin, and Diamond lattice topologies.

The stress distributions within the composite skin are presented in Figure 13. For the BCC lattice, both fidelity levels predict a similar high-stress region beneath the impact zone, although the LF model exhibits a slightly larger area of elevated stress. This behavior is consistent with the greater compliance of the beam-based lattice, which transfers a larger proportion of the impact load directly to the composite skin. In contrast, the Kelvin and Diamond lattices exhibit broader and more uniformly distributed stress fields in the HF models, indicating more efficient load redistribution through the dense lattice network. The LF models, however, predict more localized stress concentrations beneath the impact region, reflecting the reduced stiffness and simplified load transfer capability of the beam representation.

**Figure 14:**
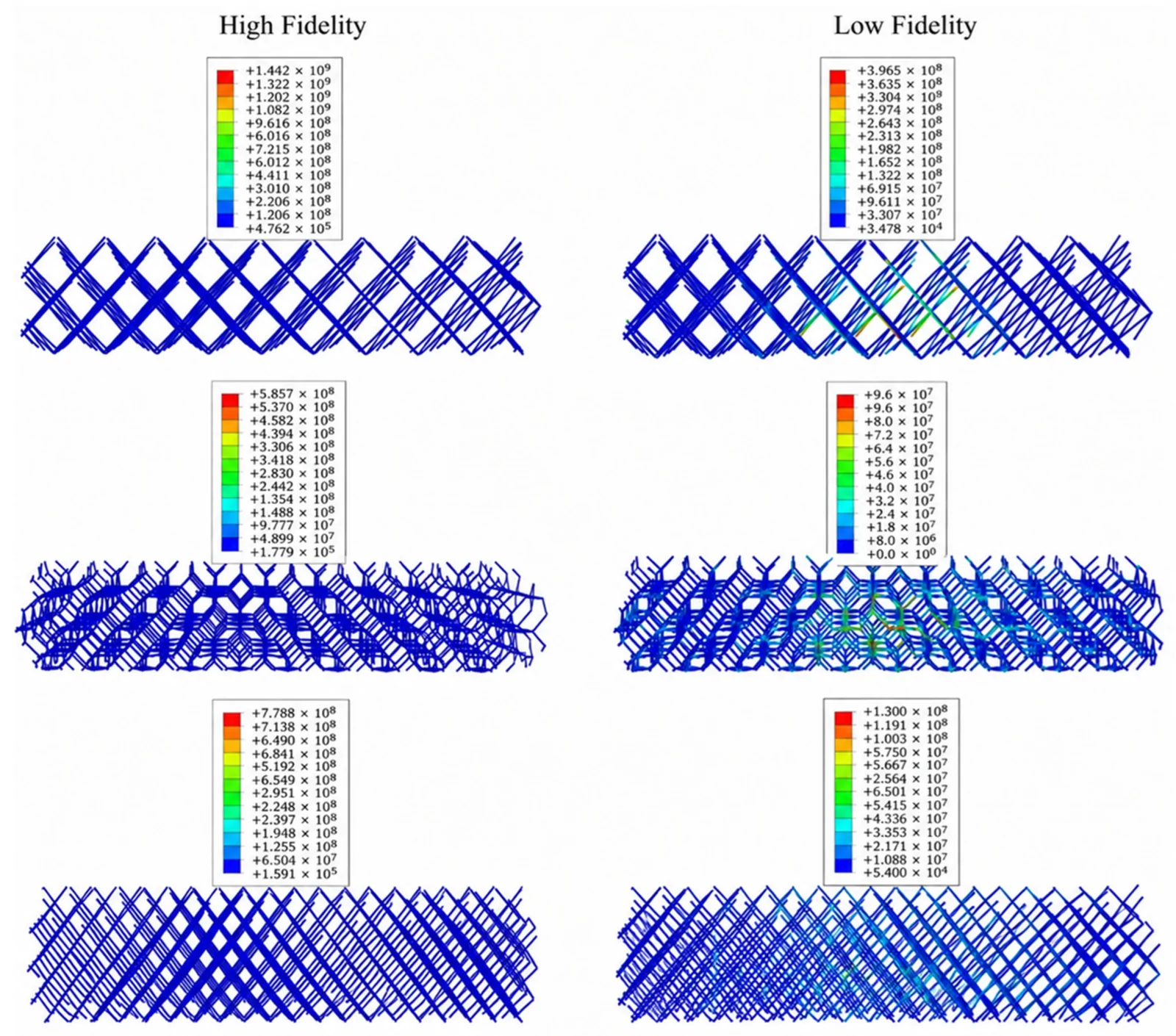
Comparison of von Mises stress (Pa) distributions within the lattice structures predicted by the HF and LF models for the BCC, Kelvin, and Diamond lattice topologies.

The differences become considerably more pronounced within the lattice structures themselves, as illustrated in Figure 14. The HF models capture distinct stress concentrations at lattice junctions where multiple struts intersect, and complex multi-axial stress states develop during impact. These highly stressed regions are, therefore, critical locations for assessing local structural integrity and the limitations of reduced-order representations. In contrast, the LF beam models produce comparatively smooth stress distributions along the struts and do not reproduce the local stress concentrations associated with three-dimensional nodal geometry. This limitation arises because the beam formulation represents each strut as a one-dimensional structural member, preventing the development of realistic joint stresses and local stress redistribution. For this reason, the stress results are used to identify differences in load transfer representation and local stress localization rather than to establish a numerical stress error metric between the two fidelity levels.

These observations provide a mechanical explanation for the larger fidelity discrepancies observed for Kelvin and Diamond in the displacement and energy analyses. A beam model can reproduce selected global response quantities while simultaneously losing local information associated with junction-level stress redistribution. Therefore, the acceptability of the LF formulation depends on the response quantity of interest: it can be appropriate for computationally efficient prediction of selected global quantities, but it should not be considered equivalent to the HF formulation when local stress concentrations or detailed junction-level load transfer are required.

Moreover, recent experimental–numerical investigations of additively manufactured Kelvin metamaterials have likewise highlighted the strong role of the Kelvin architecture in governing stiffness degradation and energy dissipation behavior under cyclic deformation. Tuninetti et al. [31] reported pronounced hysteretic energy dissipation and topology-dependent mechanical behavior in LPBF-manufactured Kelvin structures. These findings provide additional support for considering the junction-connected architecture when interpreting the present Kelvin response. In the current bird strike simulations, the larger HF-LF discrepancy observed for Kelvin, particularly in residual deformation, is consistent with the greater sensitivity of highly connected lattice architectures to local stiffness and load transfer mechanisms. However, the different loading conditions and objectives of the two studies mean that the cited work is used only as supporting evidence for the topology-dependent interpretation, rather than as a direct validation of the present impact results.

### 3.4. Computational Efficiency

Computational efficiency is an important consideration when selecting an appropriate modeling fidelity for bird strike simulations, particularly in optimization studies that require a large number of numerical analyses. The computational performance of the HF and LF models for the three lattice topologies is summarized in Table 6.

Substantial reductions in computational cost were achieved using the LF beam element models for all investigated lattice topologies. The LF models reduced the wall clock time by approximately 74% for the BCC lattice, 86% for the Kelvin lattice, and 82% for the Diamond lattice, corresponding to computational speed-up factors of 3.8×, 7.3×, and 5.6×, respectively. These results demonstrate that the computational advantage of beam models becomes increasingly significant for dense lattice architectures, where the HF models require substantially finer discretization around complex nodal regions.

The observed reduction in computational time is associated with both the reduced model size and the larger stable time increment permitted by the beam representation. In the HF models, the presence of small solid elements at lattice junctions restricts the stable time increment and, consequently, increases the total number of explicit solution increments required to complete the simulation. In contrast, the LF beam models employ one-dimensional structural elements that permit larger stable time increments while simultaneously reducing the overall model size. As a result, the computational savings become more pronounced as lattice connectivity and geometric complexity increase.

The results presented in the table indicate that LF beam models offer a highly efficient alternative for topology screening, parametric investigations, and surrogate-based optimization, where thousands of numerical evaluations may be required. However, these computational benefits should be considered together with the fidelity differences discussed in the preceding sections, particularly for dense lattice topologies where improvements in efficiency are accompanied by reduced accuracy in predicting local structural behavior.

### 3.5. Consolidated Fidelity Assessment and Modeling Implications

The results presented in Section 3.1, Section 3.2, Section 3.3 and Section 3.4 demonstrate that the discrepancy between the HF and LF formulations cannot be characterized by a single topology-independent error level. The magnitude of the discrepancy varies with both lattice topology and the structural response quantity considered. To quantify this behavior, the relative discrepancy between the LF and HF responses is defined as in Equation (1).

(1) $$\;E_{R}=\frac{R_{LF}-R_{HF}}{R_{HF}}\times 100$$

where $R_{HF}$ and $R_{LF}$ denote the corresponding responses obtained from the high- and low-fidelity models, respectively. This metric is applied to the response quantities for which direct HF/LF comparison is available.

**Figure 15:**
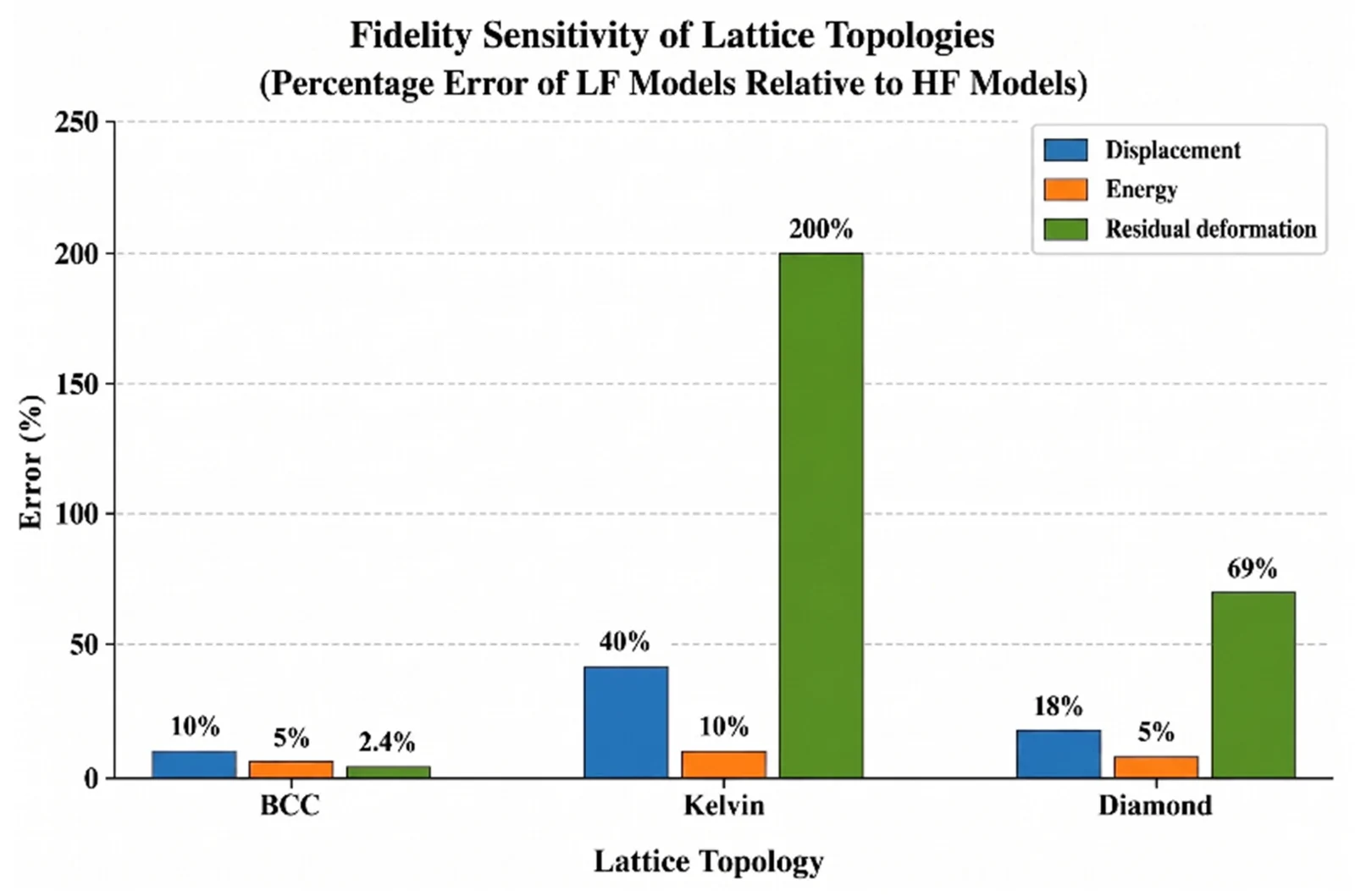
Relative discrepancy (%) of low-fidelity models relative to high-fidelity results for the investigated lattice topologies.

Table 7 and Figure 15 summarize the resulting fidelity assessment. The BCC configuration exhibits comparatively small discrepancies in the principal global response quantities, with peak displacement and total energy remaining close to the HF reference. This indicates that the beam representation is sufficiently consistent with the dominant structural response of this topology for global screening purposes. In contrast, the Kelvin configuration exhibits substantially larger discrepancies in peak and residual deformation, while the Diamond configuration shows a moderate discrepancy in peak displacement but a considerably larger difference in residual deformation. These results demonstrate that a model that appears adequate for a transient global response may not provide equivalent accuracy for the post-impact structural state.

The energy results provide a further distinction. For some configurations, the LF formulation reproduces total absorbed energy relatively closely while the partitioning of energy between the lattice and surrounding structural components differs more substantially. Consequently, agreement in a single global quantity should not be interpreted as evidence that the underlying load transfer behavior is reproduced with the same fidelity.

The stress results provide the clearest limitation of LF formulation. The HF model resolves three-dimensional stress concentrations at lattice junctions, whereas the beam formulation represents the lattice through one-dimensional members and, therefore, cannot reproduce the local stress state within the junction volume. This limitation becomes increasingly relevant for highly connected architectures, such as Kelvin and Diamond. The fidelity assessment, therefore, indicates that the suitability of the LF formulation depends on the purpose of the analysis rather than solely on the computational cost.

On this basis, the results support three practical modeling regimes. First, LF beam models are suitable for computationally efficient screening and parametric studies when the primary quantities of interest are selected global responses and the topology exhibits relatively small HF/LF discrepancies. Second, LF models should be used with caution when residual deformation or component-level energy redistribution is important, even when agreement in peak displacement or total energy appears acceptable. Third, HF solid models remain necessary when local stress concentrations, detailed junction mechanics, or other geometry-sensitive quantities are required. These guidelines provide a topology- and response-dependent basis for selecting modeling fidelity in lattice-reinforced impact structures. It is important to consider that the modeling implications identified in this study are specific to the investigated configuration and loading condition, namely, a 1 kg bird impacting the leading edge at 68.4 m/s under normal impact. Changes in impact velocity, bird mass, impact angle, or impact location may modify the relative contributions of inertia, local deformation, lattice connectivity, and load transfer, and may, therefore, alter the observed HF-LF discrepancies. Accordingly, the present fidelity assessment should be interpreted as a topology- and response-dependent comparison within the defined impact regime rather than as a universal validity criterion for beam-based lattice models.

An important implication is that fidelity should not be treated as an intrinsic binary property of a numerical formulation. Instead, it is a response-specific property of the model–topology combination. The present results, therefore, support the use of reduced-order models as purpose-dependent tools rather than as universal substitutes for detailed solid representations.

### 3.6. Comparison with Previous Studies

Table 8 compares the principal findings of the present work with representative studies reported in the literature. Previous investigations have primarily examined beam and solid element representations under quasi-static loading or have focused on demonstrating the crashworthiness benefits of lattice-reinforced aerospace structures using high-fidelity models alone. Consistent with these studies, the present work confirms that simplified beam models can reproduce the global structural response with acceptable accuracy when the deformation is governed by slender strut behavior. However, the results further demonstrate that the accuracy of low-fidelity models under bird strike loading is strongly dependent on lattice topology and the structural response quantity being evaluated. In particular, dense lattice architectures exhibit significantly greater sensitivity to modeling fidelity owing to the increased influence of three-dimensional nodal stiffness and local stress redistribution. The present work complements these studies by providing a quantitative comparison of displacement, energy absorption, stress distribution, residual deformation, and computational efficiency across multiple lattice topologies under bird strike loading.

### 3.7. Limitations and Future Work

The present study provides a controlled numerical assessment of modeling fidelity for three lattice topologies under a defined bird strike configuration. The assessment is limited to a fixed unit cell size and strut diameter, a central normal impact involving a 1 kg bird at 68.4 m/s, and the adopted numerical framework, including mass scaling and the previously validated SPH bird formulation. Boundary truncation, cell number effects, and topology-specific mesh convergence were not independently varied, while stress comparisons were primarily interpreted in terms of spatial load transfer characteristics rather than direct quantitative stress error metrics. Experimental validation of the complete lattice-reinforced leading edge and a systematic uncertainty/sensitivity assessment were also beyond the scope of the present work. Future studies will extend the framework to different lattice scales and impact conditions, experimental validation, and hybrid or adaptive beam–solid modeling strategies to further establish and broaden the applicability of the proposed fidelity assessment.

## 4. Conclusions

This study investigated the topology- and response-dependent fidelity of low-fidelity beam element models for lattice-reinforced UAV leading-edge structures subjected to bird strike impact. High-fidelity solid element and low-fidelity beam element models were evaluated under identical geometry, material properties, boundary conditions, and impact loading for BCC, Kelvin, and Diamond lattice topologies. The main conclusions are as follows:

1. Modeling fidelity depends on both lattice topology and response quantity. The BCC configuration showed relatively close agreement between HF and LF models for the principal global responses, whereas Kelvin and Diamond exhibited substantially larger discrepancies, particularly in residual deformation.
2. Global agreement does not necessarily indicate equivalent structural mechanics. For some configurations, the LF formulation reproduced total absorbed energy with relatively small discrepancy while producing different component-level energy redistribution. Similarly, comparable peak displacement predictions did not necessarily correspond to comparable residual deformation.
3. Lattice junction mechanics are a primary source of fidelity loss. The HF solid formulation resolves three-dimensional stress concentrations and load transfer behavior at lattice junctions, whereas the LF beam formulation represents the lattice through one-dimensional members. This limitation becomes more important for highly connected architectures, such as Kelvin and Diamond.
4. The computational advantage of the LF formulation is substantial. The beam-based models reduced computational time by approximately 74–86% relative to the corresponding HF models. This makes them attractive for computationally intensive screening and parametric studies, provided that the selected response quantities are not strongly sensitive to local junction mechanics.
5. Modeling fidelity should, therefore, be selected according to the purpose of the analysis. LF models can provide an efficient representation for selected global response quantities, while HF solid models remain necessary when residual deformation, component-level load transfer, local stress concentrations, or detailed junction mechanics are important.

The results, therefore, demonstrate that modeling fidelity should be selected according to the lattice topology and response quantity for the investigated configuration. The resulting assessment provides a practical basis for selecting computational fidelity for the present lattice-reinforced UAV leading-edge configuration under the investigated bird strike loading condition. Extension of these observations to different lattice scales, bird masses, impact velocities, impact angles, impact locations, or structural configurations requires further investigation.

## Figures and Tables

**Table 1:** Lattice topology and characteristics.

Parameter	BCC	Diamond	Kelvin
Unit cell size (mm)	50 × 50 × 50	50 × 50 × 50	50 × 50 × 50
Strut diameter (mm)	2	2	2
Strut aspect ratio (d/L)	0.04	0.04	0.04
Deformation mechanism	Bending-sensitive	Stretch-dominated	Multidirectional/quasi-isotropic
Connectivity	Low	Medium	High

**Table 2:** Aluminum (Al2024-T3) material properties and Johnson–Cook parameters.

Property	Symbol	Value	Unit
Density	ρ	2770	kg/m^3^
Young’s Modulus	E	74,660	MPa
Poisson’s Ratio	ν	0.30	–
Yield Stress	A	369	MPa
Hardening Constant	B	684	MPa
Hardening Exponent	n	0.73	–
Strain Rate Coefficient	C	0.0083	–

**Table 3:** CF/Pa material properties.

Property	Symbol	CF/PA	Units
Density	ρ	1160	kg/m^3^
Young’s modulus	E	13.1	GPa
Poisson’s ratio	ν	0.33	–
Ultimate tensile strength	σu	171	MPa
Fracture strain	ε_f_	0.015	–
Stress triaxiality	η	−0.33	–
Fracture energy	G_f_	2.2	kJ/m^2^

**Table 4:** High-fidelity (solid lattice) models.

Lattice Type	Lattice Nodes	Lattice Elements	Element Types	Total Nodes	Total Elements
BCC	567,063	1,328,426	424,414 C3D8 904,012 C3D4	811,491	1,472,497
Kelvin	796,221	2,174,401	517,461 C3D8 1,659,940 C3D4	1,040,649	2,318,472
Diamond	633,303	1,931,992	288,114 C3D8 1,643,878 C3D4	877,731	2,076,063

**Table 5:** Low-fidelity (beam lattice) models.

Lattice Type	Lattice Nodes	Lattice Elements	Element Type	Total Nodes	Total Elements
BCC	3085	3222	B31	247,513	147,293
Kelvin	15,446	15,000	B31	259,874	159,071
Diamond	5983	6246	B31	250,411	150,317

**Table 6:** Computational time comparison between high- and low-fidelity lattice models.

Lattice Topology	Fidelity Level	Wall Clock Time (s)	Wall Clock Time (hh:mm:ss)	Speed up (High/Low)	Time Saving (%)
BCC	High-fidelity (solid)	13,696	03:48:16	–	–
	Low fidelity (beam)	3586	00:59:46	3.8×	74%
Kelvin	High-fidelity (solid)	14,853	04:07:33	–	–
	Low fidelity (beam)	2029	00:33:49	7.3×	86%
Diamond	High-fidelity (solid)	12,508	03:28:28	–	–
	Low fidelity (beam)	2250	00:37:30	5.6×	82%

**Table 7:** Topology- and response-dependent fidelity assessment and modeling implications within the investigated scope.

Lattice Topology	Response Quantity	HF/LF Discrepancy	Fidelity Interpretation	Recommended LF Use
BCC	Peak displacement	~10%	Good agreement	Suitable for global screening
BCC	Total energy	<5%	Good agreement	Suitable for global energy assessment
BCC	Residual deformation	~3%	Good agreement	Suitable for verification
Kelvin	Peak displacement	~40%	Significant discrepancy	Use with caution
Kelvin	Residual deformation	~200%	Large discrepancy	HF recommended
Diamond	Peak displacement	~18%	Moderate discrepancy	Use with caution
Diamond	Residual deformation	~70%	Large discrepancy	HF recommended
All topologies	Total energy	<5–15%	Topology-dependent agreement	Suitable for selected global assessments
All topologies	Component-level energy	Topology-dependent	Energy partitioning differs	HF preferred
All topologies	Local stress field	Not directly equivalent	Junction-level differences	HF required

**Table 8:** Comparison of representative studies on lattice modeling fidelity and the contribution of the present work.

Reference	Structure/Application	Loading Condition	Lattice Topology	Principal Findings	Contribution Relative to the Present Study
Cao et al. (2018) [14]	SLM stainless steel lattice specimens	Quasi-static compression	Rhombic dodecahedron	Beam element models increasingly deviate from experiments as strut diameter increases because simplified beam formulations cannot accurately represent nodal constraints.	Supports the importance of nodal modeling at the unit cell level. The present study extends this concept to a complete UAV leading edge subjected to bird strike loading and demonstrates that fidelity errors are also topology dependent.
Liu et al. (2020) [15]	Node-reinforced BCC lattice	Quasi-static compression	BCC	Incorporating realistic nodal geometry significantly improves the accuracy of beam-based models, highlighting the importance of joint representation.	Confirms that nodal idealization governs fidelity loss. The present work further demonstrates that this behavior varies with lattice topology under dynamic bird strike loading.
Xia et al. (2022) [32]	Reduced-order beam model for lattice structures	Quasi-static compression and shear	Multiple topologies	Beam-based reduced-order models accurately predict the global response of slender lattice structures under static loading.	Extends these observations to high-velocity bird strike loading and demonstrates that satisfactory agreement in static analyses does not necessarily translate to dynamic impact conditions.
Annunziata et al. (2026) [33]	Hybrid beam–solid lattice model	Dynamic compression	Octagonal lattice	Hybrid modeling reproduces global structural behavior while improving local stress prediction compared with beam-only models.	Similar conclusions are obtained for dynamic loading; however, the present study evaluates three representative lattice topologies within a complete aerospace structure subjected to bird impact.
Battaglia et al. (2024) [18]	Lattice-reinforced UAV leading edge	Bird strike impact	Reference lattice design	Lattice-filled composite leading edges provide improved bird strike crashworthiness compared with conventional metallic structures.	Investigates the same engineering application but does not assess modeling fidelity or computational efficiency, which constitute the primary contribution of the present study.
Wang et al. (2025) [34]	TPMS lattice-filled aero-engine fan blade	Bird strike impact	TPMS lattice	TPMS lattices improve impact resistance and energy absorption under bird strike loading.	Confirms the benefits of lattice reinforcements in aerospace impact applications, whereas the present study focuses on topology-dependent multi-fidelity modeling and computational efficiency.

## Data Availability

The original contributions presented in this study are included in the article. Further inquiries can be directed to the corresponding authors.
